# Methylation profile of induced pluripotent stem cells generated by integration and integration-free approaches

**DOI:** 10.1016/j.dib.2018.01.061

**Published:** 2018-01-31

**Authors:** Rinat Sultanov, Olga Lebedeva, Georgij Arapidi, Maria Lagarkova, Sergei Kiselev

**Affiliations:** a Federal Research and Clinical Center of Physical Chemical Medicine of the Federal Medical and Biological Agency of the Russian Federation, Russia; b Vavilov Institute of General Genetics of the Russian Academy of Sciences, Russia

**Keywords:** Induced pluripotent stem cells, Illumina 450К Methylation BeadChip, DNA methylation, Sendai virus reprogramming, Lentiviral reprogramming

## Abstract

The genetic reprogramming technology allows generation of induced pluripotent stem cells (iPSCs) from somatic cells (Takahashi and Yamanaka, 2006) [1]. iPSCs have the ability to self-renew, and to differentiate into any type of somatic cells, and are considered as a promising tool for drug development, disease modeling, and regenerative medicine. The reprogramming factors (oct4, sox2, klf4, c-myc) can be delivered to the cell nucleus either by vectors integrating into the genome (lentiviruses, retroviruses) or by non-integrative methods (e.g., plasmids, Sendai virus, synthetic mRNAs and recombinant proteins). To evaluate the contribution of the reprogramming process isogenic system should be utilized (Shutova et al., 2016) [2]. Isogenic iPSC lines, obtained in different ways can serve the ideal system to investigate DNA methylation changes. The data presented in this article report methylation profiles for iPSC lines derived from fibroblasts of a healthy donor and PARK8-associated Parkinson's disease patient via integrating (lentiviral transfection) and non-integrating (Sendai virus infection) reprogramming using an Illumina 450K Methylation BeadChip platform. The data on DNA methylation of neurons differentiated from iPSC lines are also provided here.

**Specifications Table**

**Table:** 

Subject area	Cell biology
More specific subject area	Isogenic induced pluripotent stem cells derived from fibroblasts
Type of data	idat-files, tables with beta-values
How data was acquired	Genome methylation data was obtained using the Illumina Human Methylation BeadChip 450K platform
Data format	Raw data, analyzed data
Experimental factors	Total DNA was extracted from fibroblasts derived iPSC lines and neurons differentiated from healthy donor and Parkinson's disease patients
Experimental features	DNA methylation of iPSCs generated by integrating (lentiviral) and isogenic iPSCs generated by non-integrating (Sendai virus) methods was analyzed using Illumina 450K Methylation BeadChip and RnBeads package. DNA methylation of iPSCs derived neurons was also analyzed.
Data source location	Vavilov Institute of General Genetics of the Russian Academy of Sciences or Moscow Russia
Data accessibility	Microarray data has been deposited into the NCBI GEO database (Accession number GSE105093), https://www.ncbi.nlm.nih.gov/geo/query/acc.cgi?acc=GSE105093

**Value of the data**

● For the first time, data on DNA methylation of isogenic iPSCs obtained by integrating and non-integrating methods are reported.
● There is little data on genome-wide studies of isogenic iPSС lines. DNA methylation data of isogenic iPSCs lines, obtained by lentiviruses and Sendai virus, allow comparison of the results obtained by different reprogramming methods, including data on rare diseased iPSCs, and to combine them into large data sets.
● Epigenome-wide analysis of iPSCs often require comparison of the lines, not only with different genetic backgrounds, but also obtained in various ways. Our data will allow understanding, whether the way of reprogramming makes significant changes in the DNA methylation landscape.

## 1 Data

The data presented here originates from four iPSC lines of a healthy donor generated via integrating (IPSRG2L, IPSRG6L) and isogenic cell lines generated by non-integrating (IPSRG4S, IPSRG10S) methods at different passages [1], [2]. Two iPSC lines from the same Parkinson's disease (PD) patient with the PARK8 gene mutation were obtained by integrating (IPSPDL2.15L) and non-integrating (IPSPDL2.9S) methods and used at passage 15. Three neuronal populations enriched with tyrosine hydroxylase-positive (TH-positive) neurons differentiated from the iPSCs obtained via integrating method from a healthy donor (IPSRG2L) and two PD patients with the PARK8 gene mutation (IPSPDL1.6L and IPSPDL2.15L) were chosen for DNA methylation analysis. Cell lines are summarized in Table 1.

**Table 1:** Description of the cell lines used in the study.

Sample Name_Passage	Donor	Disease status	Cell type	iPSCs reprogramming method
IPSRG2L_DN	D1	Healthy	Differentiated neurons	–
IPSPDL2.15L_DN	P1	PD	Differentiated neurons	–
IPSPDL1.6L_DN	P2	PD	Differentiated neurons	–
IPSRG6L_p15	D1	Healthy	iPSCs	Lentiviral
IPSRG6L_p27	D1	Healthy	iPSCs	Lentiviral
IPSRG2L_p10	D1	Healthy	iPSCs	Lentiviral
IPSRG2L_p26	D1	Healthy	iPSCs	Lentiviral
IPSRG4S_p11	D1	Healthy	iPSCs	Sendai virus
IPSRG4S_p25	D1	Healthy	iPSCs	Sendai virus
IPSRG10S_p10	D1	Healthy	iPSCs	Sendai virus
IPSPDL2.15L_p15	P1	PD	iPSCs	Lentiviral
IPSPDL2.9S_p15	P1	PD	iPSCs	Sendai virus

Genomic DNA was isolated from iPSCs and differentiated neurons, converted with sodium bisulfite, and applied onto a chip according to the manufacturer's instructions. Data from the Infinium Human Methylation BeadChip 450K chip for 12 samples were deposited into the NCBI GEO database under the accession number GSE105093. Raw data with signal intensities of methylated and non-methylated samples were processed using the RnBeads package [3].

**Fig. 1:**
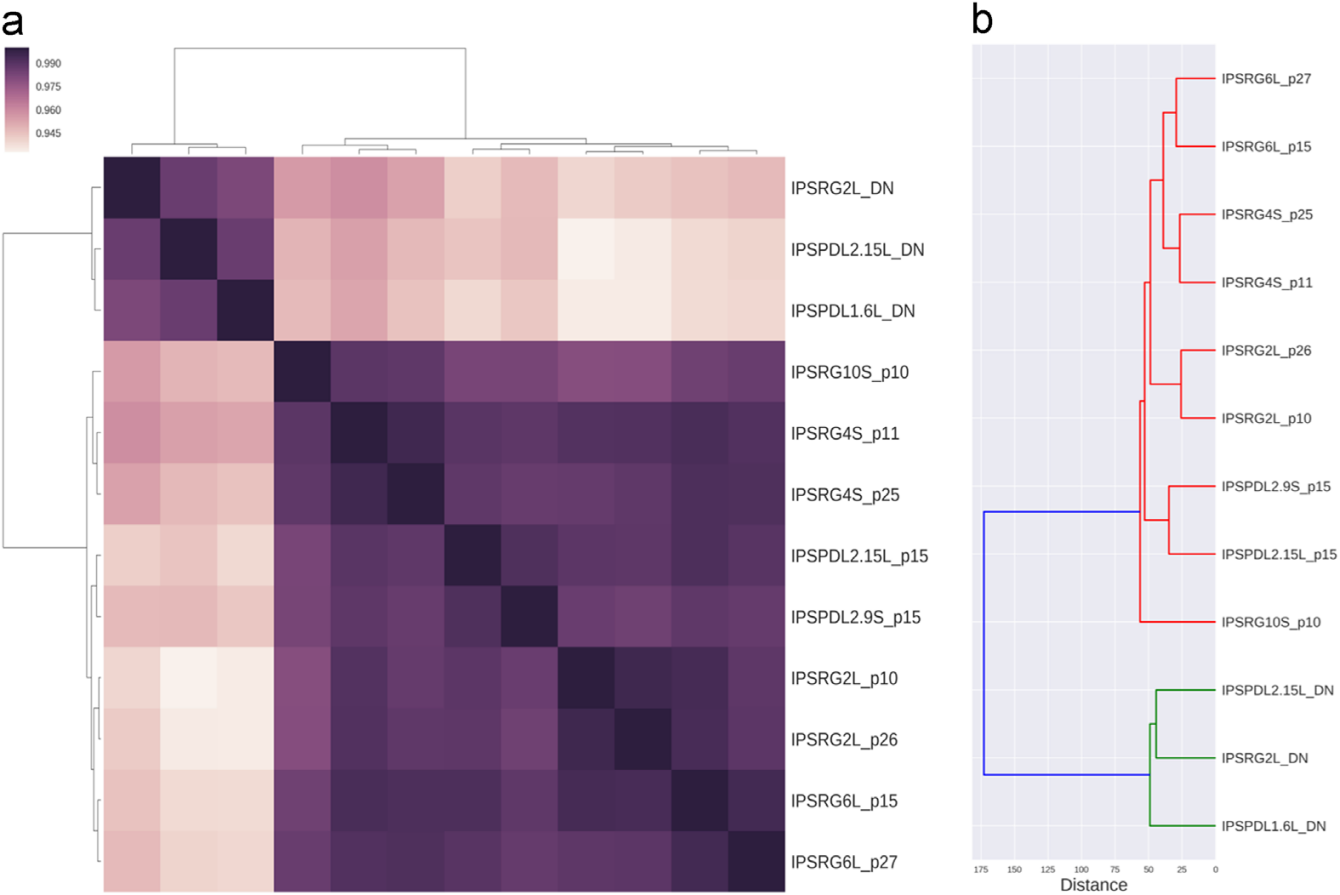
A - Spearman correlation analysis of the whole-genome DNA methylation in iPSC lines obtained by integrating and non-integrating methods and their differentiated derivatives. _pXX corresponds to passage number. _DN corresponds to neurons differentiated from the iPSCs. B - Hierarchical cluster analysis data using normalized beta-values.

Correlation analysis of the data on genome wide methylation of non-differentiated iPSC lines obtained from two different donors by two methods and their differentiated derivatives (TH-positive neurons) showed that correlation between differentiated neurons and their parent iPSCs was the least. iPSC lines grouped into two clusters according to the donor of parental fibroblasts but not reprogramming method (Fig. 1).

## 2 Experimental design, materials, and methods

### 2.1 iPSCs generation

Generation of integration free iPSC lines was described in Ref. [4], integrational iPSCs were generated as described in Ref. [5], [6].

### 2.2 Differentiation of dopaminergic neurons from iPSCs

iPSCs were grown in the medium for neuronal differentiation (DMEM/F12 medium supplemented with 2% KO SR, 1% nonessential amino acids, 2 mM L-glutamine, 50 units/ ml penicillin, 50 µg/ml streptomycin, 0.1 mM beta-mercaptoethanol 80 ng/mL Noggin, 10 µM SB431542, 2 µM dorsomorphin,) for 14 days. Then, cells were cultured for 24 more days in maturation Neurobasal Medium (Invitrogen) containing B27 supplement (Invitrogen) 2 mM L-glutamine, 10 ng/ml BDNF, and 10 ng/ml GDNF (all from PeproTech).

### 2.3 Methylation data profiling

To isolate DNA, iPSCs and neurons differentiated thereof enriched with TH-positive cells (day 38 of differentiation) were used. Both cell types were removed from the support with 0.05% trypsin solution. Trypsin was inactivated with two volumes of DMEM supplemented with 10% FBS and cells were centrifuged at 300 g for 5 min. Cell pellet was washed with PBS and centrifuged under the same conditions. DNA from the cells was isolated by phenol–chloroform extraction. The isolated DNA was subjected to bisulfite conversion, hybridization, and scanning on an iScan.

### 2.4 Methylation data analysis

To analyze the Illumina BeadChip 450K platform data, the RnBeads package was used [3]. To filter the correctly determined methylation values, the Greedycut algorithm [3] was applied to raw data. Background normalization and correction between chemical probes of different types was performed using the methylumi.noob [7] and BMIQ [8] algorithms.
